# The In Vitro Cytotoxic Effect of Elesclomol on Breast Adenocarcinoma Cells Is Enhanced by Concurrent Treatment with Glycolytic Inhibitors

**DOI:** 10.3390/cancers16234054

**Published:** 2024-12-03

**Authors:** Josephine S. Modica-Napolitano, Morgan Murray, Jacob Thibault, John-Paul Haley-Read, Lauren Nixdorf, Bridget Shanahan, Nicholas Iacovella, Carlos Reyes

**Affiliations:** Department of Biology, Merrimack College, North Andover, MA 01845, USAhaleyreadj@merrimack.edu (J.-P.H.-R.);

**Keywords:** mitochondria-targeting anticancer agents, glycolytic inhibitors, combination chemotherapy, Warburg effect, breast cancer, elesclomol, 2-deoxy-D-glucose, 3-bromopyruvate

## Abstract

**Background/Objectives:** Glycolysis and mitochondrial oxidative phosphorylation are the two major metabolic pathways for cellular ATP production. The metabolic plasticity displayed by cancer cells allows them to effectively shift between each of these pathways as a means of adapting to various growth conditions, thus ensuring their survival, proliferation and disease progression. Metabolic plasticity also provides cancer cells with the ability to circumvent many traditional monotherapies aimed at only one or the other of the major ATP-producing pathways. The purpose of this study was to determine the effectiveness of a dual treatment strategy aimed simultaneously at both pathways of ATP production in human breast cancer cells. It was hypothesized that concurrent exposure of these cells to the mitochondria-targeting chemotherapeutic agent, elesclomol, in combination with either of two glycolytic inhibitors, 2-deoxy-D-glucose or 3-bromopyruvate, would yield greater in vitro anticancer effects than those observed for any of the compounds used as a single agent. **Methods:** Cytotoxicity and clonogenic assays were employed to assess the survival and proliferation of MCF7 and MDA-MB-231 human breast adenocarcinoma cells exposed to the compounds alone and in combination. **Results:** The data obtained show that the cancer-cell-killing and antiproliferative effects of the dual treatment were significantly enhanced compared to those observed for any of the compounds alone. **Conclusions:** The results of this study are important in that they suggest the possibility of a novel and effective chemotherapeutic strategy for breast cancer cell killing.

## Simple Summary

Breast cancer cells exhibit a high degree of metabolic plasticity, allowing them to shift between glycolytic and mitochondrial ATP-producing pathways as a means of adapting to and surviving in a variety of growth conditions. Consequently, breast cancer cells exposed to a single chemotherapeutic agent that inhibits only one of these major cellular ATP-producing pathways can meet their energy demands by using the alternative pathway. This study explored a novel drug combination aimed simultaneously at both means of energy production in MCF7 and MDA-MB-231 breast adenocarcinoma cells. Our data show that dual treatment of these cells with the mitochondria-targeting anticancer agent, elesclomol, and either glycolytic inhibitor, 2-deoxy-D-glucose or 3-bromopyruvate resulted in greater cytotoxic and antiproliferative effects than those induced by any of the compounds used as a single agent. These results suggest the possibility of a novel and effective combination treatment for the enhanced cytotoxicity of breast cancer cells.

## 1. Introduction

Female breast cancer has become the most commonly diagnosed cancer and the fifth leading cause of cancer deaths worldwide [1]. With approximately 2.3 million new cases diagnosed and nearly 900,000 deaths reported in 2020 alone, the need for novel and alternative treatment strategies for this disease has never been more critical. As is commonly observed in most cancer cell types, breast cancer has both an increased energy demand compared to normal cells and a high degree of metabolic plasticity. These characteristics endow cancer cells with the ability to shift between glycolytic and mitochondrial ATP-producing pathways as a means of adapting to a variety of growth conditions thus ensuring their survival, proliferation and disease progression [2,3,4,5]. Importantly, metabolic plasticity also provides cancer cells with a means to circumvent many traditional monotherapies. As a consequence, cancer cells exposed to a single agent that inhibits only one of the major pathways for cellular ATP production can meet their energy needs by using the alternative pathway. It is of interest, therefore, to explore the possibility that combination chemotherapy aimed at both glycolytic and mitochondrial ATP-producing pathways may be an effective chemotherapeutic approach for the selective cytotoxicity of breast cancer.

**Figure 1:**
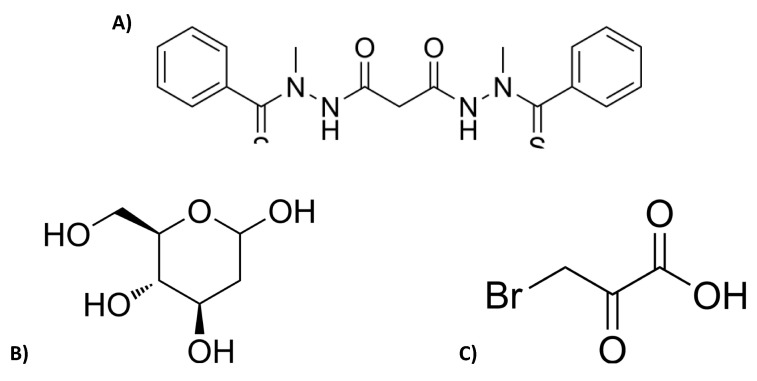
Chemical structures of (**A**) elesclomol, (**B**) 2-Deoxy-D-glucose (2 DG) and (**C**) 3-Bromopyruvate (3BP) (images obtained from MedChemExpress, https://www.medchemexpress.com/).

Elesclomol (N-malonyl-bis(N’-methyl-N’-thiobenzoylhydrazide); Figure 1A) is a mitochondria-targeting compound that exhibits selective antitumor activity against a broad range of cancer cell types in vitro, including the MCF7 and MDA-MB231 human breast adenocarcinoma cell lines [6,7,8,9,10,11,12,13]. Elesclomol has been shown to enhance reactive oxygen species (ROS) production and induce a transcriptional gene profile characteristic of an oxidative stress response [14] and to interact with the mitochondrial electron transport chain (ETC) to generate high levels of ROS within the organelle and ultimately induce cell death in yeast [15]. A more recent study conducted in this laboratory demonstrates a direct effect of elesclomol on bioenergetic function in isolated mammalian mitochondria, and suggests the possibility that the increase in ROS production and cytotoxicity induced by elseclomol may in part be due to uncoupling of mitochondrial oxidative phosphorylation and/or inhibition of ETC activity [16]. Elesclomol has also been shown to enhance the potency of known chemotherapeutic agents in human tumor models in vivo, and to display encouraging but limited therapeutic benefit in Phase I through Phase III clinical trials [8,9,10,17].

Nearly a century ago, the German biochemist Otto Warburg first observed that many cancer cells displayed enhanced glucose uptake, a high rate of glycolysis in the presence of oxygen, and an increase in lactic acid as a byproduct of the glycolytic pathway [18]. This “Warburg Effect” is now known to render many cancer cells particularly vulnerable to chemotherapeutic agents that inhibit glycolytic enzymes. For example, the glycolytic inhibitor 2-deoxy-D-glucose (2DG; Figure 1B) has shown promise as an anticancer agent in vitro and in vivo [19,20,21,22]. 2DG is a synthetic glucose analog that competes with glucose for substrate binding and phosphorylation at the first hexokinase catalyzed reaction in glycolysis. However, unlike the phosphorylated natural substrate, 2-DG-P cannot be further metabolized in the glycolytic pathway. Its accumulation within the cell results in the allosteric and competitive inhibition of hexokinase, a decrease in ATP production [22] and eventually cell death [23]. Although 2DG has been found to be highly effective in inhibiting glycolysis in cancer cells in vitro and in vivo [24,25,26] and has been tested in a number of clinical trials [23,27,28], the therapeutic potential of this compound as a single agent has not yet been demonstrated.

Another glycolytic inhibitor, 3-bromopyruvate (3BP; Figure 1C), is a structural analog of pyruvate and lactate that inhibits hexokinase-II, an isoform of the hexokinase enzyme located on the mitochondrial outer membrane. Hexokinase-II has been found to be overexpressed in cancer cells, making it an excellent candidate for selective cancer cell killing [21,29,30]. 3BP has also been shown to be a primary inhibitor of glyceraldehyde-3-phosphate dehydrogenase, the enzyme that catalyzes the sixth step in the glycolytic pathway [31]. Inhibition of glycolysis by 3BP leads to a disruption in ATP production, oxidative stress, and eventually cell death. As is the case for 2DG, despite the very promising anticancer effects exhibited by 3BP in vitro and in vivo, the clinical application of this compound as a single agent appears to be quite limited [30,32,33,34,35,36].

The purpose of this study was to explore the effectiveness of a unique in vitro combination of anticancer agents that simultaneously target the mitochondrial and glycolytic ATP-producing pathways in human breast adenocarcinoma cells. It was hypothesized that by inhibiting both pathways of cellular energy production in these cells, the anticancer effects of the combination treatment would be enhanced above and beyond that observed for any of the compounds alone. The data obtained show that dual treatment of MCF7 and MDA-MB-231 breast cancer cells with the mitochondria-targeting anticancer agent, elesclomol, and either glycolytic inhibitor, 2DG or 3BP, resulted in greater cytotoxic and antiproliferative effects on human breast adenocarcinoma cells than those induced by any of the compounds used as a single agent.

## 2. Materials and Methods

### 2.1. Materials and Reagents

Dulbecco’s Modified Eagles Medium (DMEM; catalog No. 30-2002) and Fetal bovine serum (FBS; catalog No. 30-2020) were obtained from American Type Culture Collection (Manassas, VA, USA); Thiazolyl Blue Tetrazolium Bromide (MTT; catalog No. 475989-1GM) was obtained from Millipore Sigma (Burlington, MA, USA); and dimethyl sulfoxide (DMSO; catalog No. BP231-100) was obtained from Fisher Scientific (Waltham, MA, USA). Elesclomol (catalog No. HY-12040) was obtained from MedChem Express (Monmouth Junction, NJ, USA) and made fresh on the day of experimentation at a concentration of 1 mM in DMSO; 2-deoxy-D-glucose (2DG; catalog No. AC111980050) was obtained from Fisher Scientific and made fresh on the day of experimentation at a concentration of 1 M in DMEM; and 3-bromopyruvate (3BP; catalog No. 376817-M) was obtained from Millipore Sigma and made fresh on the day of experimentation at a concentration of 50 mM in dH_2_O.

### 2.2. Cell Cultures

The human breast adenocarcinoma cell lines MCF7 and MDA-MB-231 were obtained from ATCC and grown in DMEM culture medium supplemented with 10% FBS. All cells were maintained at 37 °C in a 5% CO_2_ atmosphere.

### 2.3. Mitochondria Membrane Potential in Whole Cells

Mitochondrial membrane potential was assessed by measuring TMRE fluorescence using a Zeiss 800 confocal microscope. The cells were seeded in glass-bottom 35 mm plates (MatTek; Ashland, MA, USA) and grown to 90% confluency. The cells were washed twice with sterile PBS and incubated for 10 min with 100 nM TMRE in phenol red free Eagle’s minimum essential medium (EMEM) and two drops/mL media of the nuclear stain NucBlue Live. The cells were photographed immediately before the addition of elesclomol (t = 0) and then again after 10 min of exposure to the compound. The ionophore FCCP (carbonyl cyanide-4-(trifluoromethoxy)phenylhydrazone; 100 μM) was used as a positive control for uncoupling of oxidative phosphorylation and dissipation of mitochondrial membrane potential.

### 2.4. Glycolysis Assay

The Sigma-Aldrich Glycolysis Assay Kit (MAK439) was used according to the manufacturer’s specifications to measure L-Lactate, the glycolytic end product, in MCF7 and MDA-MB-231 cancer cell lines in the presence and absence of 2DG and 3BP. Briefly, sample wells in a 96-well plate were seeded with 100 μL of cell suspension (1 × 10^5^ cells/well) and the plate was incubated overnight at 37 °C in a 5% CO_2_ atmosphere. The next day, the original cell media were aspirated and replaced with media containing varying concentrations of 2DG and 3BP (0–1000 mM). The plate was again incubated overnight at 37 °C in a 5% CO_2_ atmosphere. After the 24 h exposure period, the working reagent for the glycolysis assay was prepared and added to each sample well. The plate was then incubated for 30 min at room temperature, after which the absorbance at 540 nm of each sample was measured using a BioTek EL800 Microplate Reader (BioTek Instruments, Inc.: Winooski, VT, USA) and L-lactate concentrations were quantified.

### 2.5. Cell Viability Assays

The MTT assay was used to assess the viability of each cell type in the absence and presence of each compound or combination of compounds. The cells were trypsinized, counted, and diluted to a concentration of 5 × 10^4^ cells/mL. Using a 96-well plate, sample wells were seeded with 100 μL of the cell suspension (0.5 × 10^3^ cells) and plates were incubated in a humidified atmosphere at 37 °C. After 24 h, varying concentrations and combinations of the test compound(s) were added and the plates were returned to the incubator for a defined exposure period (48 h). At the end of the exposure period, MTT [3-(4,5-dimethylthiazol-2-yl)-2,5-diphenyltetrazolium bromide] was added to the wells and the cells were incubated for 4 h. Solubilization buffer was then added to each well and the cells were returned to the incubator for 24 h. Using a BioTek EL800 Microplate Reader (BioTek Instruments, Inc.), the absorbance at 540 nm of each sample was measured against a blank well. A graph of the absorbance (*y*-axis) against the concentration of drug (*x*-axis) was plotted. Each treatment sample and appropriate blank was performed in triplicate for each experiment. Experiments were repeated at least three times on cultures from different platings.

### 2.6. Clonogenic Assays

The proliferative capacity and colony-forming ability of each cell type was measured in the absence and presence of each compound or combination of compounds. Cells were trypsinized, counted, diluted to a concentration of 1 × 10^3^ cells/mL, and seeded in 6-well plates with 2 mL/well of the cell suspension (i.e., 2000 cells/well). After 24 h, the media were aspirated and 2 mL/well of media containing varying concentrations of the test compound(s) were added. The cells were exposed to the compound(s) for a specified time period (48 h), after which the media were aspirated and the wells were rinsed with PBS. Each well then received 2 mL of compound-free media, and the plates were returned to their growth chambers. When the colonies in the control wells reached sufficient size for counting (approximately 7–10 days later), the plates were stained with crystal violet and the colonies in each well were counted to determine the plating efficiency (i.e., the percentage of cells seeded at subculture that give rise to colonies). Each treatment sample and appropriate blank was performed in triplicate for each experiment. The experiments were repeated three times on cultures from different platings.

### 2.7. Statistical Analysis

Statistical analysis was performed using GraphPad Prism version 9.4.1 for Mac (GraphPad Software, Inc., San Diego, CA, USA). An ordinary one-way ANOVA analysis followed by Tukey’s multiple comparison test was used to compare data generated from the control and treatment conditions. The results were expressed as a mean of a minimum of three independent experiments ± SE. Statistical significance was set at ** *p*  <  0.01, *** *p*  <  0.001, **** *p* < 0.0001.

## 3. Results

### 3.1. Elesclomol Has Direct Effects on Mitochondrial Bioenergetic Function in MCF7 and MDA-MB-231 Breast Adenocarcinoma Cells

A previous study conducted in this laboratory was the first to demonstrate that elesclomol has a direct inhibitory effect on mitochondrial bioenergetic function in isolated mammalian mitochondria and causes depolarization of the mitochondrial membrane potential in the non-tumorigenic mammalian kidney epithelial cell line, CV-1 [16]. In order to determine whether elesclomol might have a similar effect on mitochondrial membrane potential in breast adenocarcinoma cell lines, MCF7 and MDA-MB-231 cells were treated with varying concentrations of elesclomol (30–70 nM) for a constant exposure time (10 min) in the presence of Tetramethylrhodamine, ethyl ester (TMRE). TMRE is a cell-permeant, cationic fluorescent dye that is readily sequestered by active mitochondria in living cells and can be used as a monitor of mitochondrial membrane potential specifically or of mitochondrial health or dysfunction more generally.

**Figure 2:**
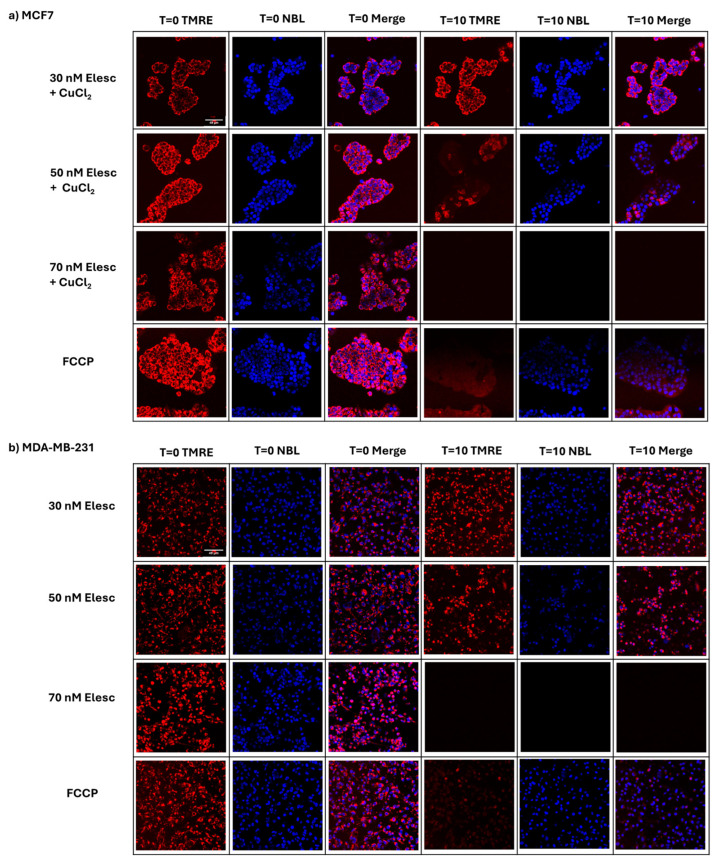
The effect of varying concentrations of elesclomol on mitochondria membrane potential in whole cells. Confocal images of control and elesclomol-treated (**a**) MCF7 and (**b**) MDA-MB-231 breast adenocarcinoma cells were observed after staining with the membrane-potential-dependent fluorescent dye TMRE and the nuclear stain NucBlue Live (NBL) prior to treatment (t = 0) and 10 min after treatment (t = 10) with elesclomol (Elesc). Over the same time period with FCCP, a proton ionophore and known uncoupler of oxidative phosphorylation, DMSO, the solvent for elesclomol, and CuCl_2_, an additive required for MCF7 cells, were used as controls. The images shown are representative of three separate experiments, in which a minimum of three cells/field and three fields/slide were imaged for each treatment plate at 20× magnification using a Zeiss confocal microscope. Image processing was performed using FIJI/ImageJ software (version 1.54g). Brightness was adjusted to improve clarity.

The data presented in Figure 2 demonstrate that after 10 min of exposure to elesclomol, a significant and concentration-dependent decrease in TMRE fluorescence was observed in MCF7 and MDA-MB-231 cells when compared to the control (t = 0), indicating a concentration-dependent depolarization of the mitochondrial membrane potential. Specifically, in both cell lines, exposure to a concentration of 30 nM elesclomol induced no obvious change in TMRE fluorescence intensity, indicating no effect on the mitochondrial membrane potential. However, exposure to 50 nM elesclomol caused a partial decrease in TMRE staining and exposure to 70 nM elesclomol caused a complete dissipation of TMRE staining, indicating a partial loss and complete depolarization of the mitochondrial membrane potential, respectively. Additionally, at 70 nM elesclomol, the NucBlue stain was also lost, suggesting complete degradation of nuclear DNA at a high concentration of the compound.

The biologically active form of elesclomol is a deprotonated copper chelate [37]. Upon therapeutic administration in vivo, the chelate forms when elesclomol acquires Cu^2+^ in the bloodstream. However, in vitro studies have indicated that the addition of CuCl_2_ to the incubation medium concurrent with elesclomol exposure was required to induce cytotoxicity [16,37]. In this study, in MCF7 cells, the addition of equimolar concentrations of elesclomol and CuCl_2_ to the incubation medium was required to induce depolarization of the mitochondrial membrane potential. This is similar to what we have shown previously in CV-1 cells [16]. In contrast, depolarization of the membrane potential in MDA-MB-231 cells did not require the addition of CuCl_2_, suggesting the possibility of higher levels of endogenous copper in this cell line. Of further note, the concentration of elesclomol shown to cause complete uncoupling of the mitochondrial membrane potential in MCF7 and MDA-MB-231 cells (70 nM) is much lower than that which we have previously shown to induce this same effect in CV-1 cells (40 μM) [16]. This suggests that the breast adenocarcinoma cell lines may be more sensitive than the nontumorigenic cell line to the anti-mitochondrial and subsequent cytotoxic effects of the compound.

### 3.2. 2DG and 3BP Inhibit Glycolysis in MCF7 and MDA-MB-231 Cells

In order to confirm that 2DG and 3BP inhibit glycolysis in MCF7 and MDA-MB-231 cells and to assess their effective inhibitory concentration range for this study, a spectrophotometric assay was conducted in which the amount of the glycolytic end product L-lactate was measured in the presence and absence of each compound. In this assay, the L-lactate that was secreted into the cell media after exposure to each of the compounds was quantified using a coupled reaction involving the lactate dehydrogenase catalyzed oxidation of L-lactate. This biochemical reaction generates pyruvate and NADH, which subsequently reduces a formazan dye. The intensity of the reduced dye, measured at 565 nm, is directly proportional to the L-lactate concentration in the sample, which, in turn, is directly proportional to the glycolytic rate of the cells.

**Figure 3:**
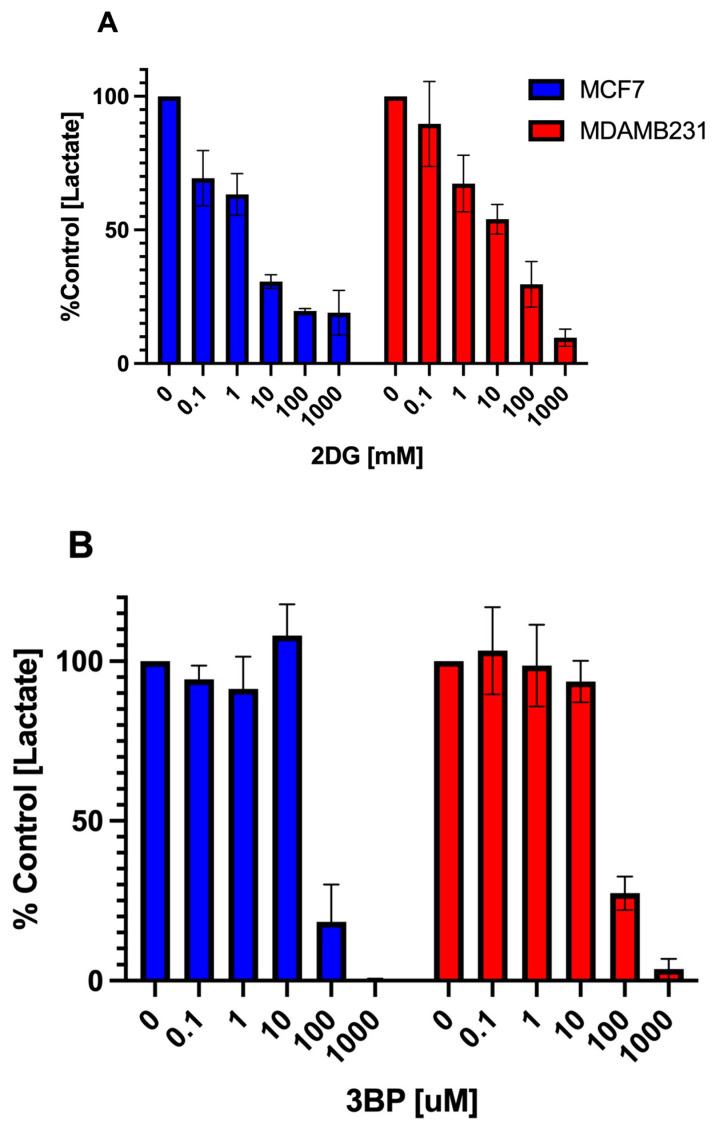
The effect of 2DG and 3BP on glycolytic activity in breast cancer cells. A glycolysis assay was used to determine the inhibitory effect of (**A**) 2DG and (**B**) 3BP on L-lactate production in MCF7 and MDA-MB-231 cells after a 24 h exposure period. The data points represent the average values obtained for three separate experiments, +/− S.E.

As shown in Figure 3A, 2DG induced a graded, concentration-dependent inhibition of L-lactate production in both MCF7 and MDA-MB-231 cells over the range of concentrations tested (0–1000 mM). The data presented in Figure 3B show that inhibition of L-lactate production by 3BP occurred only at the higher end of the concentration range tested (i.e., between 100 and 1000 mM) in both cell lines.

### 3.3. The Effect of Elesclomol, 2DG, and 3BP as Single Agents on the Viability and Proliferative Capacity of Breast Cancer Cells

**Figure 4:**
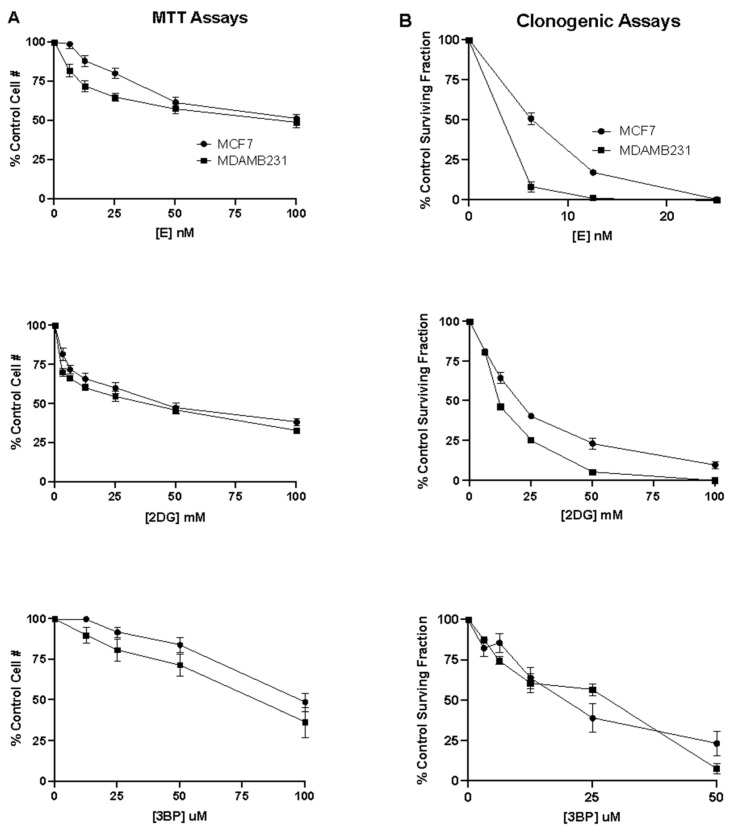
The effect of elesclomol, 2DG and 3BP as single agents on breast cancer cell viability and proliferation. (**A**) MTT assays were performed to assess the cytotoxic effect of elesclomol, 2DG, and 3BP as single agents on MCF7 and MDA-MB-231 cell lines. (**B**) Clonogenic assays were performed to assess the colony-forming ability of MCF-7 or MDA-MB-231 breast cancer cells after exposure to varying concentrations of elesclomol, 2DG or 3BP for 48 h. The data points in each plot represent the average values obtained for 3–6 separate experiments, +/− S.E.

The MTT colorimetric assay, which monitors cell viability, was used to measure the cytotoxic effect of breast cancer cells in response to elesclomol, 2DG, or 3BP used as single agents. This was accomplished by measuring the cell number in the absence and presence of varying concentrations of elesclomol, 2DG, or 3BP for a defined exposure period. The results in Figure 4A show that all three compounds induce concentration-dependent cytotoxicity in breast cancer cells. It is of interest to note that for each compound, the extent of the cytotoxic response was comparable in the MCF7 and MDA-MB-231 cell lines. The results show that elesclomol is a much more potent cytotoxic agent than either of the glycolytic inhibitors. In both cell lines, the average concentration required to kill half of the exposed cell population (LC_50_) at 48 h treatment was found to be approximately 100 nM, whereas the LC_50_ for 2DG and 3BP was found to be 50 mM and 100 uM, respectively.

The clonogenic assay, which evaluates the ability of a single cell to grow into a colony, was then used to assess the long-term survival and proliferative capacity of breast cancer cells in response to elesclomol, 2DG, or 3BP used as single agents. This was accomplished by measuring colony formation in the absence and presence of varying concentrations of each test compound for a defined exposure period. The results in Figure 4B show that all three compounds induce a concentration-dependent inhibition of colony formation in both cell lines. As was the case for the cytotoxicity assay, elesclomol was observed to be the most potent of all three compounds, with an LC_50_ for colony formation in the nanomolar range compared to the millimolar range for 2DG and the micromolar range for 3BP. Interestingly, the clonogenic assay results demonstrate a similar inhibitory effect of 3BP on colony formation in both cell lines. However, the MDA-MB-231 cells were found to be nearly 2-fold more sensitive than the MCF7 cells to the anti-proliferative effects of elesclomol and 2DG. As expected, the results also show that all three compounds induce anti-proliferative effects at concentrations much lower than those required to induce cytotoxic effects.

### 3.4. The Effect of Elesclomol, 3BP and 2DG in Combination on the Viability and Proliferative Capacity of Breast Cancer Cells

**Figure 5:**
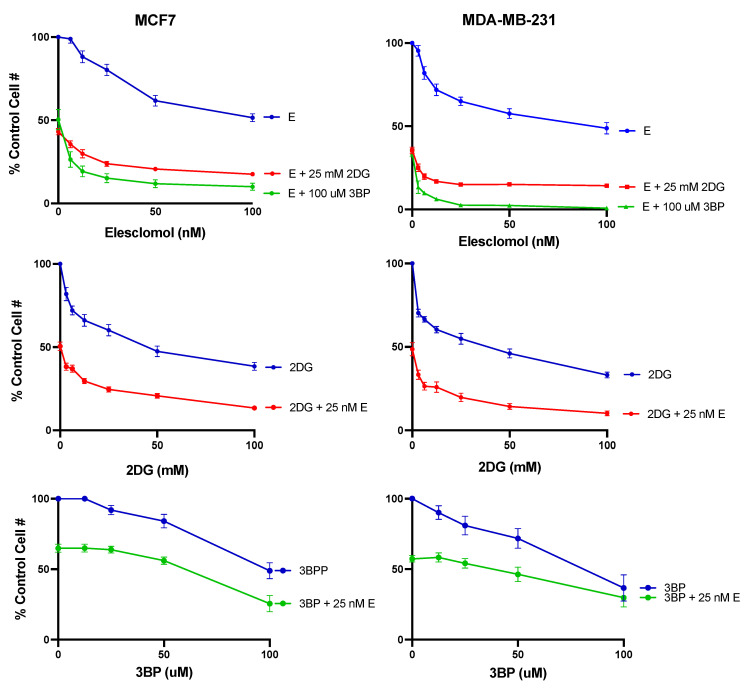
The cytotoxic effect of test compounds on breast cancer cells. MTT assays were performed to compare the cytotoxic effect of elesclomol, 2DG, and 3BP alone and in combination at the concentrations indicated in MCF7 or MDA-MB-231 human breast cancer cells. The data points represent the average values obtained for 3–6 separate experiments, +/− S.E.

The MTT colorimetric assay was then used to measure the cytotoxic effect of breast cancer cells in response to elesclomol in combination with 2DG or 3BP. The results presented in Figure 5 show that when the mitochondria-targeting agent elesclomol was combined with either glycolytic inhibitor, the cytotoxic effect on both breast cancer cell lines was significantly enhanced compared to that observed for any of the compounds used as single agents. This was the case whether the experimental conditions involved varying elesclomol concentrations in the presence of a constant concentration of glycolytic inhibitor or varying glycolytic inhibitor in the presence of a constant concentration of elesclomol.

**Figure 6:**
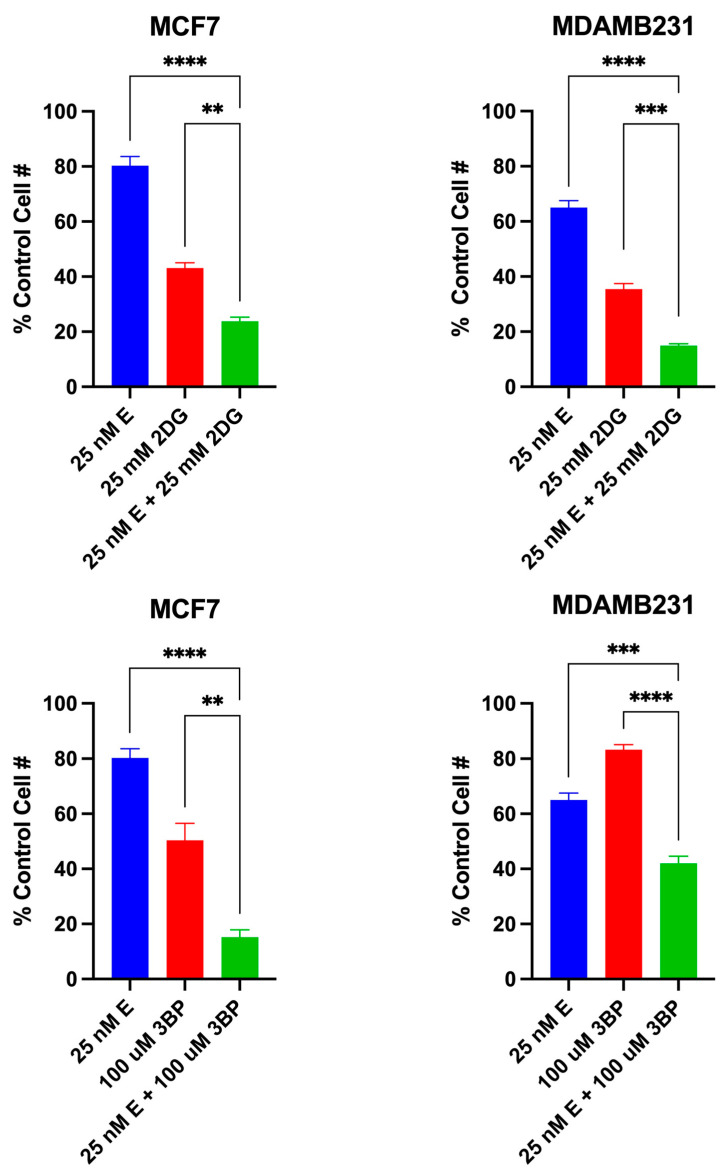
The additive cytotoxic effect of the test combinations. The data points represent the average values obtained for 3–6 separate experiments, +/− S.E. Statistical analysis was performed using an ordinary one-way ANOVA analysis followed by Tukey’s multiple comparisons test. The threshold for statistical significance was set to ** *p*  <  0.01, *** *p*  <  0.001, **** *p* < 0.0001.

The data presented in Figure 6 are from another series of MTT assays using specific compound concentrations that were chosen based on the results from the dose–response curves in Figure 5. The data show that in both MCF7 and MDAMB231 cell lines, the combined effect of elesclomol with either 2DG or 3BP is equal to the sum of the effects of the two drugs acting independently. This apparent additive cytotoxic effect is the expected result when each drug in a therapeutic combination has a different target with overlapping actions (i.e., the mitochondrial and glycolytic ATP-producing pathways). As noted in Figure 6, the difference in the cytotoxic effect induced by elesclomol, 2DG or 3BP as single agents versus that induced by the elesclomol in combination with 2DG or 3BP is highly significant.

**Figure 7:**
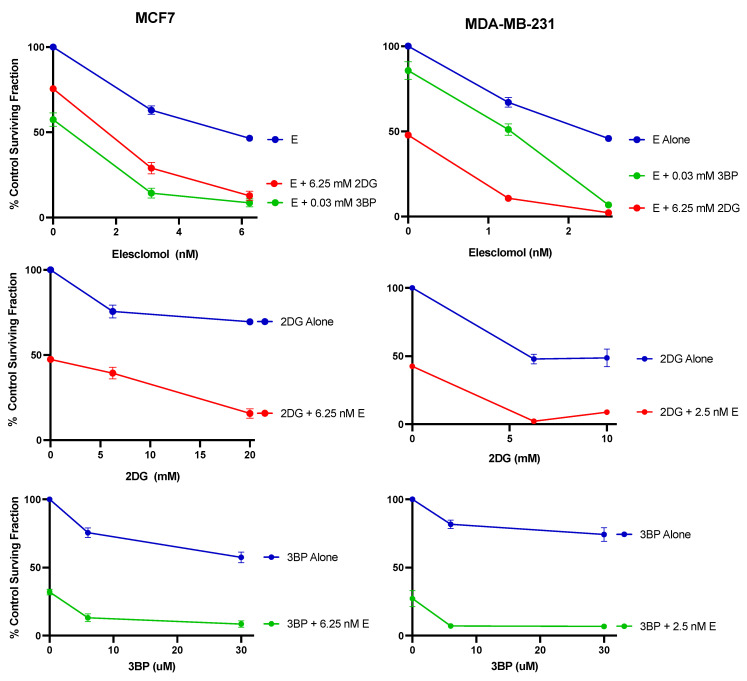
The antiproliferative effect of test compounds on breast cancer cells. Clonogenic assays were performed to compare the colony-forming ability of MCF7 or MDA-MB-231 human breast cancer cells exposed to varying concentrations of elesclomol, 2DG, and 3BP alone and in combination at the concentrations indicated. The data points represent the average values obtained for 3–4 separate experiments, +/− S.E.

**Figure 8:**
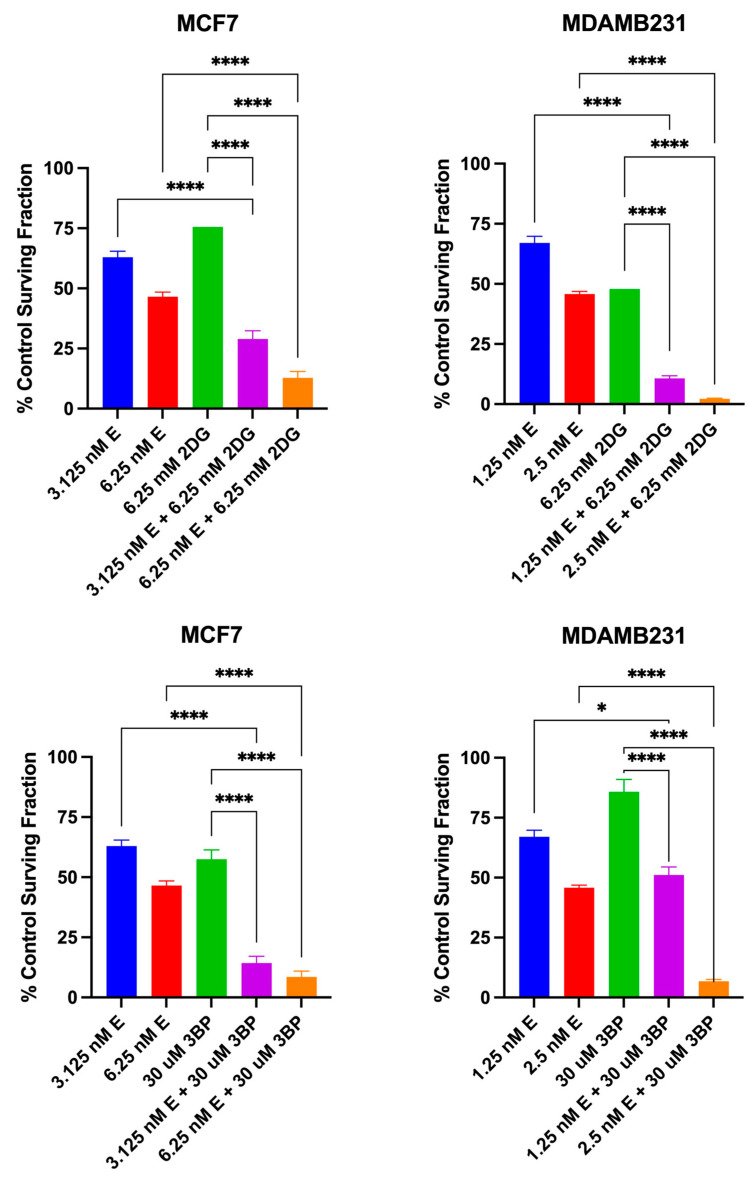
The additive antiproliferative effect of the test combinations. The data points represent the average values obtained for three separate experiments, +/− S.E. Statistical analysis was performed using an ordinary one-way ANOVA analysis followed by Tukey’s multiple comparisons test. The threshold for statistical significance was set to * *p* < 0.05, **** *p* < 0.0001.

The results of the clonogenic assay presented in Figure 7 show that when elesclomol was combined with either 2DG or 3BP, the anti-proliferative effect on both breast cancer cell lines is significantly greater than that seen for any of the compounds used as single agents. Consistent with the cytotoxic effects of combination treatment, this was the case whether the experimental conditions involved varying elesclomol concentrations in the presence of a constant concentration of glycolytic inhibitor, or varying glycolytic inhibitor in the presence of a constant concentration of elesclomol. The data presented in Figure 8 are from another series of clonogenic assays using specific compound concentrations that were chosen based on the results from the dose response curves in Figure 7. Similar to that shown for the combined cytotoxic effect, the results indicate that in both cell lines, the combined antiproliferative effect of elesclomol with either 2DG or 3BP is additive and significantly different from that induced by any of the compounds as single agents.

## 4. Discussion

As noted previously, despite quite promising pre-clinical evaluations of elesclomol, 2DG or 3BP, their therapeutic potential as single agents has yet to be realized. However, the results obtained in this study and others suggest that a more promising clinical application may very well be in combination therapy. Interestingly, the efficacy of the glycolytic inhibitors 2DG and 3BP used in combination with other anticancer compounds, including those targeting the mitochondria, has been explored previously with encouraging results. For example, the combination of 2DG and metformin was shown to inhibit both glycolysis and mitochondrial respiration in prostate cancer cells, leading to a severe depletion in cellular ATP [38], and this cytotoxic effect was much greater than that observed with either drug alone. Furthermore, the 2DG and metformin combination induced only moderate cytotoxicity in the normal prostate control cells. In another study, the in vitro combination of 2DG and metformin was found to decrease cellular ATP and induce significant cell death in a variety of human cancer cell lines [39]. However, the inhibition of glycolysis with 2DG alone, even at clinically achievable doses and prolonged incubation, was insufficient to cause significant cell death. In the same study, the combination of 2DG and metformin was also demonstrated to inhibit tumor growth and metastasis in mouse xenograft tumor models injected with MDA-MB-231 breast cancer cells in vivo [39]. Interestingly, the commonly prescribed antidiabetic drug metformin has been shown to induce mitochondria-targeting effects by inhibiting oxygen consumption in colon cancer cells [40] and mitochondrial respiratory complex I and in hepatocytes [41]. More recently, the dual targeting of cellular bioenergetics with 2DG and another mitochondrial respiratory complex I inhibitor, MDIVI-1, induced a pronounced inhibition of colony formation when compared to 2DG alone [4]. Notably, the ETC respiratory enzyme complex I is especially susceptible to the inhibitory effect of elesclomol [16]. The results of our study support and extend these findings of enhanced anticancer activity when 2DG is used in combination with other mitochondria-targeting agents, especially those that inhibit ETC complex I.

With regard to 3BP, cell proliferation analysis showed that the combination of this glycolytic inhibitor with rapamycin synergistically inhibited the proliferation of neuroblastoma cells [42]. It is of interest to note that the protein kinase inhibitor rapamycin significantly affects mitochondrial health through the depolarization of the mitochondrial membrane potential and alterations in mitochondrial dynamics [43]. 3BP was also shown to synergistically augment the in vitro and in vivo anticancer activity of methyl jasmonate [44], another anticancer compound that exerts its effects through multiple mechanisms, including dissipation of the mitochondrial membrane potential, ATP depletion, and activation of the apoptotic process [45]. The results of our study show an enhanced cytotoxic efficacy against breast cancer cells when either glycolytic inhibitor, 2DG or 3BP, is used in combination with the mitochondria-targeting anticancer agent, elesclomol. Additionally, in light of the aforementioned studies with 2DG and 3BP, our results suggest more generally that a combination of anticancer compounds aimed at both glycolytic and mitochondrial pathways for ATP production may be an effective chemotherapeutic approach for the selective cytotoxicity of cancer cells.

As is suggested for the glycolytic inhibitors, combination therapy may prove to be a more promising chemotherapeutic strategy for elesclomol as well. For example, in a Phase I study of patients with refractory solid tumors, elesclomol used in combination with paclitaxel was shown to enhance the anticancer effects of paclitaxel alone [9]. In a Phase IIB randomized study of patients with metastatic melanoma, the elesclomol/paclitaxel combination doubled the median progression-free survival (PFS) of patients when compared to paclitaxel as a single agent [46]. In the follow-up Phase III clinical study, the elesclomol/paclitaxel combination treatment revealed a statistically significant improvement in median PFS in patients with normal baseline lactate dehydrogenase (LDH), yet presented minimal benefit and even adverse effects in patients with high serum LDH [11]. Interestingly, high LDH levels have been associated with tumor hypoxia and cancer cell populations that rely more heavily on glycolysis than on oxidative phosphorylation for ATP generation [47]. From the results of our study, it is reasonable to hypothesize that the addition of a glycolytic inhibitor, such as 2DG or 3BP, may improve the clinical outcome of the elesclomol/paclitaxel treatment in patients with high serum LDH.

Breast cancer is a heterogeneous disease, with different subtypes favoring different metabolic pathways based on the presence or absence of specific mutations [4]. In our study, the combination treatment of elesclomol with 2DG or 3BP was tested in two different human breast cancer subtypes. MCF7 is an estrogen-receptor-positive cell line [48,49,50]. This subtype of breast cancer has been found to be more dependent on oxidative phosphorylation than on glycolysis for their energy supply [51,52]. In contrast, MDA-MB-231 is a triple-negative breast cancer (TNBC), a specific subtype that lacks the estrogen receptor, progesterone receptor, and human epidermal growth factor receptor 2 [53,54]. It has been shown that TNBC subtypes rely mainly on glycolysis compared to oxidative phosphorylation for their energy supply [51,55,56]. The TNBC subtype is highly aggressive, invasive, prone to relapse, insensitive to endocrine therapy or HER2 treatment [57], and has a poor clinical prognosis. Consequently, the development of new TNBC treatment strategies has become an urgent clinical need. The results of our study are important in that they show TNBC MDA-MB-231 cells to be just as sensitive as MCF7 cells to the cytotoxic and antiproliferative effects of either the elesclomol plus 2DG or elesclomol plus 3BP combination.

A persistent challenge in cancer therapy is to find ways to improve the efficacy and selectivity of a therapeutic compound while minimizing its systemic toxicity and treatment-limiting side effects. As demonstrated in this study, combination chemotherapy that simultaneously targets both mitochondrial and glycolytic ATP-producing pathways in cancer cells may be one means to achieve this end. The results of this study clearly demonstrate that elesclomol in combination with either 2DG or 3BP significantly enhances the cytotoxic and antiproliferative effects induced by any of the compounds used as single agents. Furthermore, because this combination targets different metabolic pathways, potential additional benefits include a high probability of non-overlapping toxic side effects, inhibition of drug resistance to either of the single agents, and increased efficacy against heterogeneous tumors comprising some cells resistant to the mitochondria-targeting agent and some cells resistant to the glycolytic inhibitor.

The purpose of our study was to test the specific hypothesis that concurrent exposure of MCF7 and MDA-MB-231 breast cancer cells to the mitochondria-targeting chemotherapeutic agent, elesclomol, in combination with either of two glycolytic inhibitors, 2DG or 3BP, would yield greater in vitro anticancer effects than those observed for any of the compounds used as a single agent. The data obtained support this hypothesis. Although there are obvious limitations imposed by the particular in vitro approach used in this study, it is hoped that our findings will encourage further experimentation in this area. For example, it is important to elucidate the detailed molecular mechanism of action of the elesclomol plus 2DG/3BP combination treatment in MCF7 and MD-MB-231 breast cancer cells, and to determine whether the enhanced cytotoxic and antiproliferative effects of this specific combination might be extended to other breast cancer cell lines or even to other cancer cell types. Of course, in vivo studies, both experimental and clinical trials, will be necessary to evaluate the full benefit of this unique dual treatment strategy and its potential for future therapeutic application. Furthermore, it would be of interest to determine whether other combinations of mitochondria-targeting compounds and glycolytic inhibitors might yield enhanced anticancer effects equal to or greater than those observed in this study, suggesting a more broadly applicable chemotherapeutic strategy for cancer.

## 5. Conclusions

This study investigated the effectiveness of a dual treatment strategy aimed simultaneously at both mitochondrial and glycolytic pathways of ATP production in MCF7 and MDA-MB-231 human breast cancer cells. The data obtained demonstrate that concurrent exposure of these cells to the mitochondria-targeting chemotherapeutic agent, elesclomol, in combination with either of two glycolytic inhibitors, 2DG or 3BP, yields significantly greater in vitro anticancer effects than those observed for any of the compounds used as a single agent. The results of this study are important in that they suggest the possibility of a novel and effective chemotherapeutic strategy for breast cancer cell killing.

## Data Availability

The data presented in this study are available in this article.
